# Phacoemulsification and IOL-Implantation without Using Viscoelastics: Combined Modeling of Thermo Fluid Dynamics, Clinical Outcomes, and Endothelial Cell Density

**DOI:** 10.3390/s21072399

**Published:** 2021-03-30

**Authors:** Nikola Goles, Marko Nerancic, Sanja Konjik, Brigitte Pajic-Eggspuehler, Bojan Pajic, Zeljka Cvejic

**Affiliations:** 1 Department of Physics, Faculty of Sciences, University of Novi Sad, 21000 Novi Sad, Serbia; df.nikola.goles@student.pmf.uns.ac.rs (N.G.); df.marko.nerancic@student.pmf.uns.ac.rs (M.N.); bpajic@datacomm.ch (B.P.); 2 Department of Mathematics and Informatics, Faculty of Sciences, University of Novi Sad, 21000 Novi Sad, Serbia; sanja.konjik@dmi.uns.ac.rs; 3 Eye Clinic Orasis, Swiss Eye Research Foundation, 5734 Reinach AG, Switzerland; brigitte.pajic@orasis.ch; 4 Division of Ophthalmology, Department of Clinical Neurosciences, Geneva University Hospitals, 1205 Geneva, Switzerland; 5 Faculty of Medicine, University of Geneva, 1211 Geneva, Switzerland; 6 Faculty of Medicine of the Military Medical Academy, University of Defense, 11000 Belgrade, Serbia

**Keywords:** phacoemulsification, viscoelastics, corneal endothelial cells, intraocular pressure (IOP), thermo fluid dynamics, numerical simulation

## Abstract

Phacoemulsification is a widely used surgical method in cataract surgery with a high energy ultrasound source. The viscoelastic is considered to be tissue protective. The aim of this study is to investigate during surgery the impact of using viscoelastic versus no viscoelastic on clinical outcomes, potential complications and effect on endothelial cell density. The study group included 64 patients, who were subjected to phacoemulsification using balanced salt solution (BSS). Control group consisted of 62 patients, who underwent phacoemulsification using Hyaloronic acid 1% Healon 1%. Student’s *t*-test was applied for statistical analysis. The simulations of temperature changes during phacoemulsification were performed by COMSOL Multiphysics software. In the BSS group, a mean endothelial cell loss (ECL) of 4.5% was measured one month postoperatively, while in the Healon group ECL was 5.3%. Data analysis showed no significant difference in ECL between the groups (Student’s *t*-test, *p* = 0.8). No significant difference was observed in endothelial cell morphology and IOP between the two groups pre- and postoperatively (all *p* > 0.05). The modeling of thermo fluid dynamics showed that the heating of the cornea is slightly less when Healon was used as irrigation fluid. The phacoemulsification technique can be performed by an experienced surgeon with viscoelastics or continuous anterior chamber (AC) irrigation on the same level of safety regarding endothelial cell damage, providing equally satisfying clinical outcomes.

## 1. Introduction

The mean endothelial cell count at birth varies significantly between 3500 and 7500 cells/mm^2^ [1,2,3]. At the beginning of life, the rate of endothelial cell loss is physiologically high, and this curve flattens out during the course of life. Bourne et al. calculated an annual mean endothelial cell loss rate of approximately 0.6% in adulthood [4]. The average endothelial cell count in adulthood is estimated to range between 1800–2500 cells/mm^2^ [5,6,7].

The absolute endothelial cell count is not the only factor for ensuring the proper corneal endothelial function. Endothelial cell morphology, as evaluated by the examination of endothelial cell size and shape, is of paramount importance for the reliable assessment of the endothelial quality and the documentation of potential endothelial cell damage [8,9]. Critical endothelial cell count for the preservation of the endothelial function and the maintenance of corneal stromal transparency is reported to vary significantly between 300 and 800 cells/mm^2^ [10,11].

Although phacoemulsification is considered to be a low-risk surgical procedure, the following factors could be associated intraoperatively with further endothelial cell damage: direct instrument contact, liquid turbulence, whirl lens fragments due to turbulence caused by the irrigation system, excessive ultrasound energy, prolonged surgery duration, pharmacological affection, and toxic damage [12,13,14,15,16,17]. The effects of irrigating pressure and temperature rise in the anterior chamber could cause corneal endothelial damage. The ultrasound energy at the phaco tip induces the formation of free hydroxyl radicals in the aqueous solution of the anterior chamber, which can further damage the corneal endothelium. Nevertheless, mechanically induced traumas during the surgery represent the most frequent cause of endothelial cell loss in phacoemulsification [18]. Viscoelastic solutions are used in modern cataract surgery for the prevention of corneal endothelial cell loss.

We also tried to establish the relation between an increase in temperature caused by phacoemulsification with/without viscoelastics, and potential damage on the corneal endothelium. The endothelium is sensitive to thermal damage and needs to deal with possible thermal stress resulting from temperature rise during ultrasound exposure. For this purpose, the temperature distribution during phacoemulsification was modeled and the mechanisms that act as heat sources were considered. Laminar flow module and heat transfer module were considered as physics problems in COMSOL Multiphysics. Continuity, energy, and Navier-Stokes equations were simultaneously solved for steady-state and time-dependent conditions in the anterior chamber. Boussinesq approximation was also used to reduce the complexity of density change due to the temperature difference.

In this context, the aim of this study was to analyze the clinical outcomes and safety profile of phacoemulsification without use of viscoelastics.

## 2. Materials and Methods

### 2.1. Medical Study Part

This was retrospective case series comparative study. The study was approved by the Ethics Committee in Novi Sad and bears the number 34-08.18. The study group included 47 (32 females/15 males) patients subjected to phacoemulsification using balanced salt solution (BSS, Alcon, Fort Worth, TX, USA) and continuous anterior chamber irrigation instead of viscoelastics. The control group consisted of 45 (30 females/15 males) patients who underwent phacoemulsification using Healon (Alcon, Fort Worth, TX, USA). A CataRhex 3 (Oertli Instrumente AG, Berneck, Switzerland) was used as a phacoemulsification device for the study. The device was equipped with an easyPhaco^®^ tip, which enables targeted emulsification through a focused axial release of ultrasound energy. Both groups were age- and gender-matched. The same experienced surgeon performed all surgical procedures. Written patient consent was obtained by all participants. Eligible for inclusion in the study were patients with age between 40–80 years, and age-related cataract up to nuclear grade IV. Exclusion criteria included: pre-existing glaucoma, underlying pseudoexfoliation, history of uveitis, history of previous intraocular surgery, diabetes mellitus type I, corneal endothelial dystrophy/degeneration, endothelial cell density below 1400 mm/cm^2^, anterior chamber depth below 2.5 mm, and dilated pupil size below 7 mm. Intraoperative exclusion criteria were total surgical time exceeding 15 minutes, total phaco time exceeding 10 s, as well as intraoperative complications.

Mean age of patients was 71.2 years (range: 44–88 years) in the BSS group and 71.2 years (range: 40–91 years) in the control group. Pre-operative ophthalmological examination included manifest refraction, distance visual acuity (best-corrected and uncorrected) measured by Snellen optotype charts, measurement of intraocular pressure (IOP) by Goldmann applanation tonometry, measurement of corneal endothelial cell count and evaluation of endothelial cell morphology by non-contact endothelial microscopy, and slit-lamp examination of the anterior and posterior segment. Nuclear cataract was graded using the Lens Opacities Classification System III (LOCS III) [19]. The IOL power was calculated using the SRK/T formula (IOL-Master, Carl Zeiss Meditec, Oberkochen, Germany). 

Patients were allocated alternately to the BSS group or the Healon group according to the chronological order by which they were first assessed. All patients were evaluated preoperatively and 1 day, 1 week and 1 month after surgery. IOP was measured additionally 6 h after surgery.

#### 2.1.1. Non-Contact Corneal Endothelium Microscopy

Non-contact corneal endothelium microscopy (Tomey EM-3000) was used for estimation of corneal endothelium cell count and investigation of the endothelial morphology. Endothelial cells were counted in an area of 860 µm × 650 µm. Counting frame was manually adjusted for the evaluation of cells divided by the left and lower edge. Cell shape specification was estimated in % of evaluated cells; based on the cell shape of quadrangular, pentagonal, hexagonal, heptagonal and other cells. Cell area specification was stated in % of evaluated cells based on cell size; divided into cells bigger than 700 μm^2^, 600–700 μm^2^, 500–600 μm^2^, 400–500 μm^2^, 300–400 μm^2^, 200–300 μm^2^, smaller than 200 μm^2^. Qualitative endothelial changes (polymegathism and pleomorphism) were assessed with an image analysis procedure. The analyzed endothelial cell number was defined as endothelial cell density (ECD-measured in cells/mm^2^). The mean endothelial cell size was described by the average cell area (AVE-measured in µm^2^). The variation coefficient CV was received by division of the cell size standard deviation with the mean endothelial cell size. For obtaining size in percent, the coefficient had to be multiplied by 100 (SD/AVE × 100). The percentage of hexagonal cells is described by the parameter 6A. The hexagonal cell (apex) percentage and the average dispersion of endothelial cell size (area) were imaged by a diagram.

#### 2.1.2. Surgery Method with BSS

The anesthesia was performed by intracameral of Rapidocain 1% and Carbostesin 0.5%. Two incisions were placed at 10 o’clock and 2 o’clock. The anterior chamber was maintained with the irrigation terminal while performing a circular capsulorhexis with a 25 G needle. A clear corneal approach with a width of 2.2 mm was performed at 11 o’clock with consecutive hydrodissection. The lens nucleus was removed by phacoemulsification and the cortex by bimanual irrigation/aspiration (I/A) system. An acrylic IOL (type Nidek NS 60YG) was implanted by an injector in the posterior chamber during irrigation. The IOL was centered with the aspiration unit. Intracameral application of 0.25 mL Carbachol (Miostat^®^) and 0.1 mm Cefuroxim (50 mg) (Aprokam^®^) was performed at the end of the surgery.

#### 2.1.3. Surgery Method with Healon

In this group, a circular capsulorhexis was performed under the protection of Healon with a 25 G needle. The IOL was implanted with the use of Healon in the bag with a consecutive bimanual aspiration of Healon and an irrigation/aspiration (I/A) system. All other surgical steps were identical to the procedure followed in the BSS group.

#### 2.1.4. Statistical Analysis

IBM SPSS Statistics version 22.0 (IBM Corp., Armonk, NY, USA) was applied for the statistical calculations. Kolmogorov-Smirnov and Shapiro-Wilk tests were performed to examine data sets to determine whether they are parametric (normal distribution) or non-parametric. A data set is considered normally distributed if *p* > 0.05. The parametric data sets are further analyzed with Student’s *t*-test and the non-parametric data sets with the Friedmann test. Significance is present if *p* < 0.05. 

### 2.2. Modeling of Temperature Dissipation in the Anterior Chamber of the Eye during Phacoemulsification

**Figure 1:**
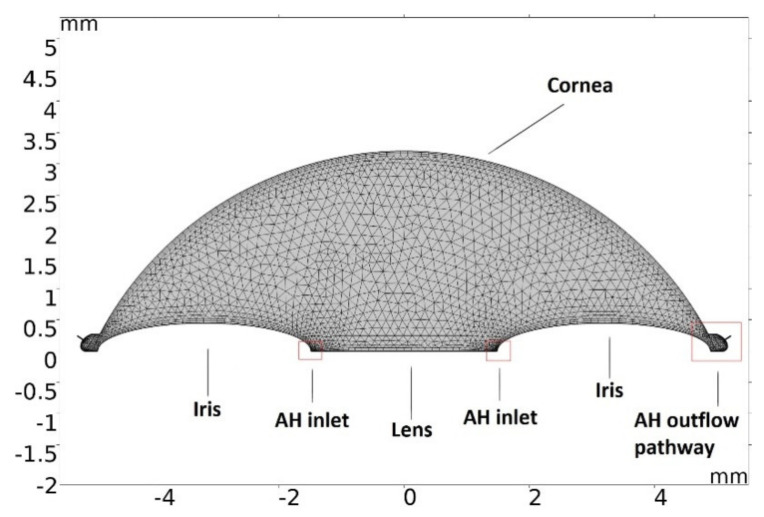
COMSOL generated mesh.

**Figure 2:**
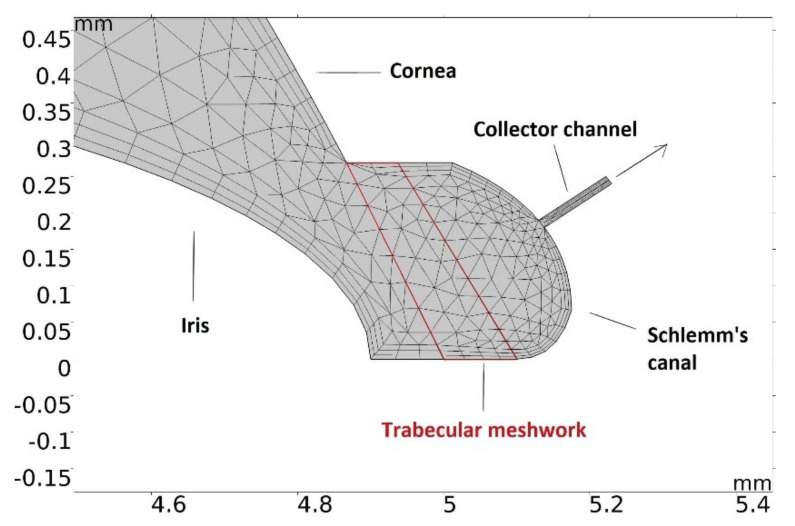
AH outflow pathway detail.

To obtain the temperature profile in the anterior chamber (AC) of the eye, during phacoemulsification with/without viscoelastics, a two-dimensional finite element model was constructed. COMSOL Multiphysics^®^ (http://www.comsol.com/, accessed on 30 January 2021) is a finite element analysis software package used to solve coupled differential equations of laminar fluid flow and heat transport. A physics-optimized mesh consisting of 4981 triangular elements was automatically generated for the simulation (Figure 1 and Figure 2). 

The relevant dimensions of the observed domain are given in next order: anterior chamber (AC) length—10 mm, AC depth—3.2 mm, pupil length—2.98 mm, iris thickness—0.45 mm [20]. At the outer rim of the AC, a system consisting of the porous trabecular meshwork (TM) and the Schlemm’s canal (SC) was implemented as the outflow pathway for the aqueous humor (AH). Parameters relevant to this construction, as well as the constants related to the AH, are given in the following order: dynamic viscosity $\mu =7.5\cdot 10^{-4}\;\mathrm{Pas}$, density $\rho =998\;{\mathrm{kgm}}^{-3}$, thermal conductivity $k=0.58\;{\mathrm{Wm}}^{-1}K^{-1}$, heat capacity at constant pressure $C_{p}$ is $\;4.2\;\;{\mathrm{Jkg}}^{-1}K^{-1}$, coefficient of thermal expansion α is $\;3\cdot 10^{-4}K^{-1}$ [21,22]. Corresponding values for BSS and Healon were taken as: $\mu_{BSS}\;=\;1.02\cdot 10^{-3}\mathrm{Pas}$; $\mu_{H}\;=\;200\mathrm{Pas}$; $C_{pBSS}\;=\;2.5\;\;{\mathrm{Jkg}}^{-1}K^{-1}$; $C_{pH}\;=\;2.5\;\;{\mathrm{Jkg}}^{-1}K^{-1}$; $k_{BSS}\;=\;0.65\;{\mathrm{Wm}}^{-1}K^{-1}$ and $k_{H}\;=\;0.47\;{\mathrm{Wm}}^{-1}K^{-1}$.

Since the main resistance to AH outflow is localized within the TM with the juxtacanalicular meshwork, average TM length—85 μm, for TM width—300 μm, TM porosity $\epsilon_{p}$—0.1, TM permeability $\kappa -5\cdot 10^{-15}m^{2}$, and a value of 163 μm for SC length [23], were taken as boundary conditions. The AH inlets consisted of two 20 μm long parts on each side of the pupil, bordering with the iris. The initial natural inflow rate was also taken from [23]. The outer wall of the AC was kept at a temperature of 27 °C, while the rest of the borders were fixed at a core body temperature of 36.5 °C. Venous pressure at the SC outlet was assumed to be 10.5 mmHg [23].

To simulate the heating induced by the ultrasonic impulse, a temporary condition of a boundary heat source representing the probe tip with a diameter 2.2 mm placed at 0.25 mm above the lens surface was added in the duration of the first 5 s of the simulation. The combined effect of the radiant, as well as viscous friction heating, was taken to total 0.66 W, 1.2 W, 2.4 W and 3 W throughout the duration of the impulse, spread out over the tip of the probe. The relevant boundary condition can be given by the following equation:

(1) n⋅∇T=Q

where ***n*** is the unit vector perpendicular to the boundary and *Q* is the total heat flux.

For the duration of the impulse, as well as in the seconds following it when the temperature dissipation was observed, a transient solution was obtained for equations governing incompressible laminar fluid flow in porous media are as follows:

(2) $$\frac{1}{\epsilon_{p}}\rho \frac{\partial u}{\partial t}+\frac{1}{\epsilon_{p}}\rho \left(u\cdot \nabla \right)u\frac{1}{\epsilon_{p}}=\nabla \cdot \left(-p2I+K\right)-\left(\mu \kappa^{-1}+\beta_{F}\left|u\right|+\frac{Q_{m}}{\epsilon_{p}^{2}}\right)u+F$$

where:

(3) $$K=\mu \frac{1}{\epsilon_{p}}\left(\nabla u+{\left(\nabla u\right)}^{T}\right)-\frac{2}{3}\mu \frac{1}{\epsilon_{p}}\left(\nabla \cdot u\right)I$$

(4) $$\rho \nabla \cdot u=Q_{m}$$

Here, the independent variables obtained by the solver were the velocity field u and pressure p; I is the identity matrix, and F represents any volumetric force acting on the domain, such as gravity.

The previous three equations were coupled with the heat transport equation in laminar fluids, in its time-dependent form:

(5) $$\rho C_{p}\frac{\partial T}{\partial t}+\rho C_{p}u\cdot \nabla T+\nabla \cdot \left(-k\nabla T\right)=Q+q_{0}+Q_{p}+Q_{vd}$$

where T is the temperature field and Q is the heat source (sink) flux, which in the case of this simulation encompasses viscous dissipation, pressure work and boundary heat source representing the surgical instrument. 

Since the only porous medium domain is located in the TM, for the rest of the simulated chamber Equations (2)–(4) are greatly simplified:

(6) $$\rho \frac{\partial u}{\partial t}+\rho \left(u\cdot \nabla \right)u=\nabla \cdot \left(-p2I+K\right)+F$$

where:

(7) $$K=\mu \left(\nabla u+{\left(\nabla u\right)}^{T}\right)$$

(8) ρ∇⋅u=0

The buoyancy effects, through which convection inside the AC occurs, were accounted for by using the Boussinesq approximation in the external volumetric force term of Equations (2) and (6):

(9) $$F=\rho \alpha g\left(T_{ref}-T\right)$$

where $T_{ref}$ is taken as the ambient temperature and α is the AH coefficient of thermal expansion.

In general, no analytical solutions exist for nonlinear models described by previous equations. The discretization governing equations and solving by Computational Fluid Dynamics (CFD) is the most common approach to fluid dynamics problems. There are various discretization techniques such as the finite difference method and the finite volume method, recently presented in [24,25], that deal with heat and mass transfer in the human eye. In this paper, the finite element method was used as a discretization technique. The solution domain is subdivided into a finite number of elements; the governing equation is solved for each element and then the overall solution is obtained by “assembly”.

Serving as the initial state of the system prior to the application of the ultrasound impulse, a stationary solution was obtained for the aforementioned equations by neglecting the time-dependent terms (i.e., the terms containing time derivatives of the sought quantities). Dealing with the case of the time-dependent solution, the equations were solved for 15 or 30 s after the onset of the impulse, depending on the time needed for the system to relax to the steady state, with a time step of 0.02 s. Since the time step of 0.02 s was small enough, this allowed for a better estimation of the pattern of temperature and velocity distribution in AC. Several assumptions were made in order to simplify the calculations: (1) Self heating rate of the probe is negligible; (2) Around the phacoemulsification probe exists a thin layer of incompressible fluid flow; (3) The heat source due to friction from the probe is the main cause of the increase in ocular temperature; (4) The frictional heat generation takes place in the entire lens because the phaco probe is repeatedly moved across the lens surface and spends equal amounts of time at each point of the entire lens.

The solver, solving algorithm and tolerances are automatically assigned by COMSOL, based on the physics modules used in the model so that the solution is convergent. In this particular problem, the assigned direct solver for each step of the was PARADISO, with physics-controlled tolerances in the time-dependent part, and a predefined relative tolerance of $10^{-3}$ in the case of the steady-state solution.

## 3. Results

### 3.1. Modeling of Temperature and Velocity Distribution in the Anterior Chamber of the Eye

The convection from the surface of the cornea has been neglected in the modeling process, because it did not play a significant role in heat transfer within the eye during the process of phacoemulsification. Namely, the outer surface of the cornea is constantly exposed to electromagnetic radiation and when the process is stopped, convection still occurs due to natural air flow and evaporation which results in tears. The aforementioned processes provide us a baseline in steady state for comparison with cases where heating effects, due to phacoemulsification, are taken into account. Furthermore, it enables an assessment of possible damage to the endothelial cells caused by an increase in internal temperature after heating and it possibly depends on the type of irrigation liquids.

**Figure 3:**
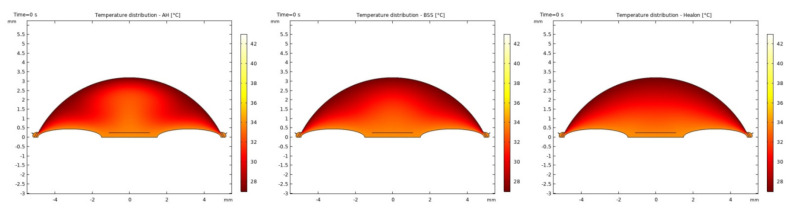
Initial temperature distributions with AH, BSS and Healon.

**Figure 4:**
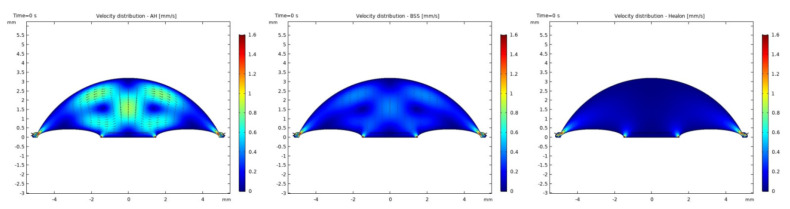
Initial velocity distributions with AH, BSS and Healon.

**Figure 5:**
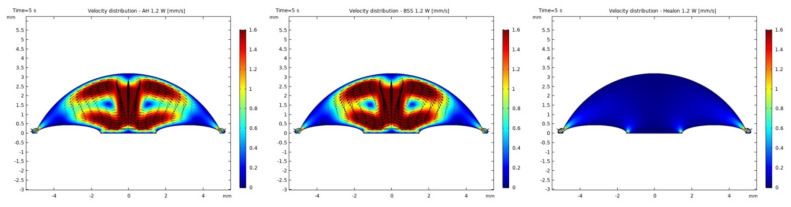
Velocity distributions with AH, BSS and Healon after 5 s of 1.2 W impulse.

**Figure 6:**
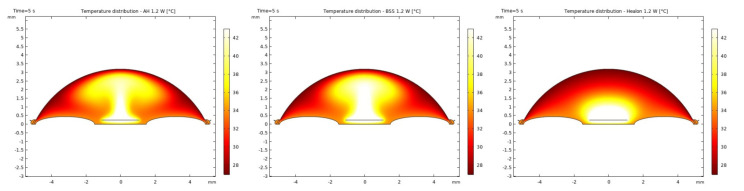
Temperature distributions with AH, BSS and Healon after 5 s of 1.2 W impulse.

**Figure 7:**
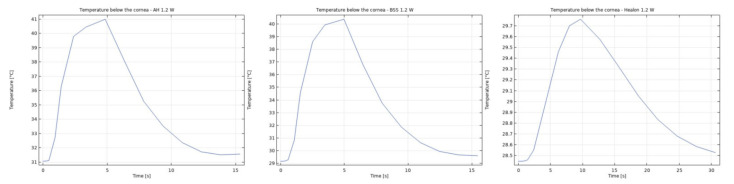
Temperature at the point probe with AH, BSS and Healon after 5 s of 1.2 W impulse.

**Figure 8:**
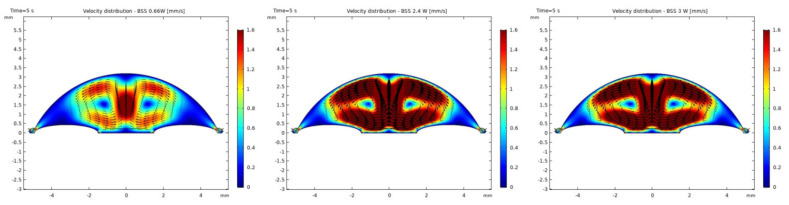
Velocity distributions with BSS after 5 s of 0.66 W, 2.4 W and 3 W impulse.

**Figure 9:**
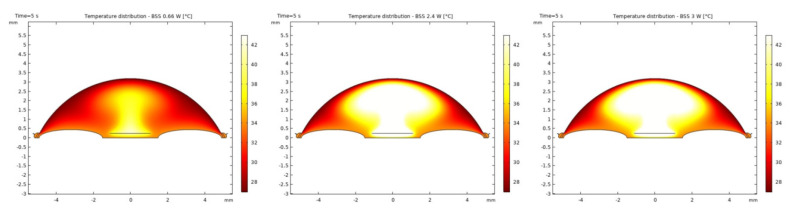
Temperature distributions with BSS after 5 s of 0.66 W, 2.4 W and 3 W impulse.

**Figure 10:**
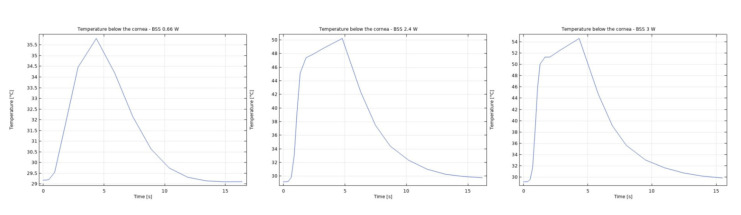
Temperature at the point probe with BSS after 5 s of 0.66 W, 2.4 W and 3 W impulse.

**Figure 11:**
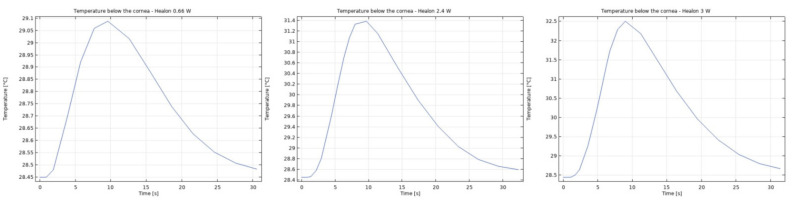
Temperature at the point probe with Healon after 5 s of 0.66 W, 2.4 W and 3 W impulse.

It was observed that the system returns to the initial steady-state solution in 10–25 s after the end of the applied impulse. The potential risk of a harmful temperature rise is emphasized by the calculated maximum temperature rise of more than 20 °C in certain conditions at a point probe placed 0.6 mm below the center of the cornea. The initial temperature and velocity distributions, before the application of the ultrasound, are given on Figure 3 and Figure 4. Snapshots of the velocity and temperature profiles in the whole domain, as well as the temperature at the point probe throughout the impulse, were taken for each of the studied heat flux values and irrigation fluids. The velocity profile graphs represent values of the velocity of fluids inside the simulated domain in each point throughout the chamber, with warmer colors on the graph representing faster movement of fluid. Color scale bar indicates velocity in mm/s and streamlines show the velocity field. These results are represented in the following figures (Figure 5, Figure 6, Figure 7, Figure 8, Figure 9, Figure 10 and Figure 11).

The ultrasounds warm the surroundings of the phacoemulsification probe in the first 5 s of the simulation. The greatest temperature increase occurred at the lens and declined toward the cornea. At the location of the aforementioned point probe, temperature increases up to 54 °C in certain conditions. These results are in good agreement with values published in [26,27].

### 3.2. Results in Medical Part

There was no significant difference regarding the age between the BSS group (71.2 ± 9.7 years) and the Healon group (71.2 ± 10.6 years) (Student’s *t*-test, *p* = 0.99).

Regarding corneal endothelial cell count, a Shapiro-Wilk and a Kolmogorov-Smirnov test were performed for the analysis of the groups. A *p* > 0.05 was found for all groups and time points. In this sense the data set is normally distributed and for the further statistical calculation parametric tests are used, the ANOVA test where more than two groups are compared and Student’s *t*-test when two groups were analyzed together.

**Figure 12:**
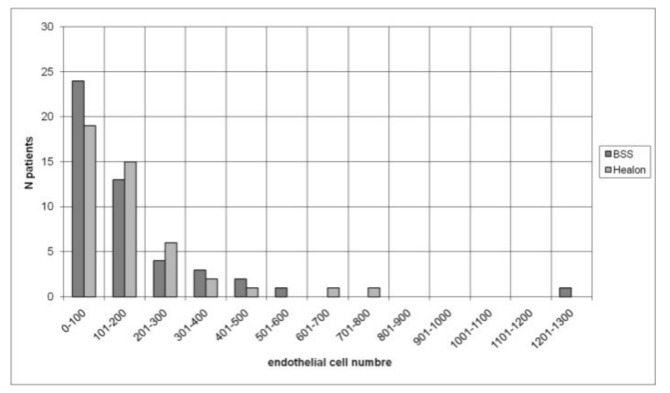
Number of patients via endothelial cell loss.

In the BSS group, the mean value of the endothelial cell count preoperatively was 2334 ± 436 cells/mm^2^. On the first postoperative day 2250 ± 472 cells/mm^2^ could be detected, after the first postoperative week 2211 ± 485 cells/mm^2^ and one month after surgery 2228 ± 471 cells/mm^2^. Comparing the values preoperatively and one month postoperatively, the mean endothelial cell loss in the BSS group was 106.2 ± 226 cells/mm^2^, which corresponds to a loss of 4.5%. In the Healon group, the mean endothelial cell count preoperatively was 2307 ± 427 cells/mm^2^. On the first postoperative day 2243 ± 435 cells/mm^2^, one week and one month after surgery 2195 ± 452 cells/mm^2^ and 2185 ±389 cells/mm^2^, respectively, could be determined. Compared to the preoperative value, the mean endothelial cell count in the Healon group decreased by 122.2 ± 207 cells/mm^2^ one month after surgery, which corresponds to a loss of 5.3% (Figure 12). There was no significant difference in corneal endothelial cell loss between the BSS and Healon groups preoperatively (*p* = 0.68), one day (*p* = 0.61), one week (*p* = 0.84) and one month postoperatively (*p* = 0.72). ANOVA test was used to calculate the progression of corneal endothelial cell count within the groups. It was found that in the BSS group the corneal endothelial cell loss from preoperative to 1 day postoperative was significant (*p* = 0.042) and from 1 day postoperative no significance was found, respectively *p* > 0.05. In the Healon group the corneal endothelial cell loss from preoperative to 1 day postoperative was significant (*p* = 0.48). From the first day onwards, no further significance was found (*p* > 0.05). 

Preoperative mean AVG was 457.7 ± 252 μm^2^ (range 170.1 to 942.6 m^2^) in the BSS group and 452.3 ± 126 μm^2^ (range 158.2 to 933.9 μm^2^) in the Healon group. Coefficient of variation (CV) was 34.7 ± 5.9 in the BSS group, with a percent of hexagonal cells (6A) of 60.4%. Coefficient of variation (CV) was 34.9 ± 5.1 in the Healon group with a percent of hexagonal cells (6A) of 58.8% (Table 1). There were no significant differences between the two groups (Student’s *t*-test, *p* > 0.05).

The mean endothelial cell area pattern and shape at first postoperative day is shown in Table 2. There were no statistically significant differences at any time between the two groups (all *p* > 0.05).

Four weeks after cataract surgery, the number of hexagonal cells decreased by 3.5% in the BSS group and by 3.2% in the Healon group, but the difference in the decrease of hexagonal cells (pleomorphism) between the two groups was not significant (*p* = 0.05). Coefficient of variation of cell size (polymegathism) was approximately 1.8% in the BSS group and 2% in the Healon group (*p* = 0.8).

The trend shows almost no statistical correlation between the preoperative corneal endothelial cell count and the preoperative age of the patient, calculated with the Student *t*-test (*p* = 0.059).

The age of the patient played a significant role in relation to corneal endothelial cell count loss one day (*p* = 0.04), one week (*p* = 0.003) and one month postoperatively (*p* = 0.049). The older the patient, the greater the endothelial cell loss after cataract surgery.

The preoperative BCVA was 0.39 ± 0.14 in the Healon group and 0.30 ± 0.14 in the BSS group, which was not statistically significant (*p* = 0.33). One day postoperatively, the BCVA was 0.68 ± 0.19 in the Healon group and 0.72 ± 0.18 in the BSS group. There was no significance between the groups (*p* = 0.479), but the BCVA increase was statistically significant within the Healon group (*p* = 0.001) and the BSS group (*p* = 0.003). One week postoperatively, BCVA was 0.88 ± 0.17 in the Healon group and 0.74 ± 0.18 in the BSS group, which is not statistically significant (*p* = 0.118). Within the group, the BCVA increase was significant in the Healon group (*p* = 0.001) and in the BSS group (*p* = 0.002) compared to the preoperative values. One month postoperative BCVA in the Healon group was 0.92 ± 0.22 and in the BSS group 0.88 ± 0.18 which is not statistically significant (*p* = 0.321). However, the BCVA increase within the Healon group (*p* = 0.001) and the BSS group (*p* = 0.006) is significant.

**Figure 13:**
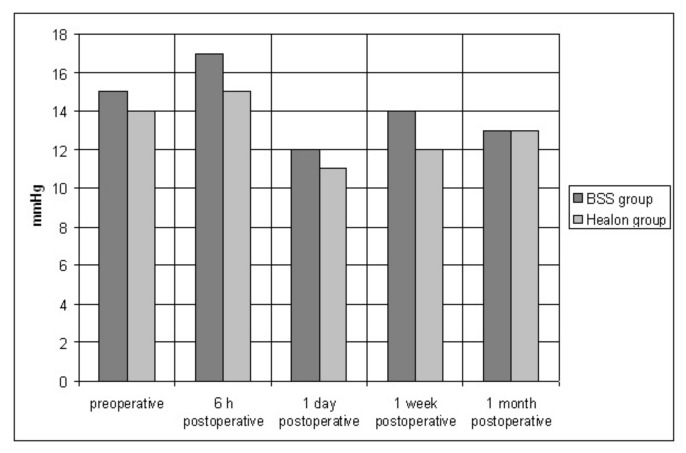
Mean IOP, preoperative and postoperative (6 h, 1 day, 1 week and 1 month).

The preoperative mean IOP was 15.2 ± 3.8 mmHg (range 6.0 to 24.0 mmHg) in the BSS group and 13.9 ± 2.7 mmHg (range 7.0 to 19.0 mmHg) in the Healon group. The difference was not significant (*p* = 0.05) (Figure 13).

IOP recordings 6 h after surgery, at first postoperative day, 1 week and four weeks after surgery, are shown in Figure 13. There were no statistically significant differences at any time between the two groups (all *p* > 0.05).

The Phaco time was 1.75 ± 0.89 s for the Healon group and 1.62 ± 0.60 s for the BSS group (Student T test *p* = 0.777). The effective phaco time (EPT) was 1.40 ± 0.71s in the Healon group and 1.31 ± 0.49 in the BSS group (Student T test *p* = 0.396). According to the Lens Opacities Classification System III (LOCS III), the cataract density grad was 2.16 ± 0.95 in the Healon group and 2.40 ± 0.81 in the BSS group (Friedmann test *p* = 0.153). No statistical difference was found between the groups in Phaco time, EPT or cataract density. Surgery time was 6.54 ± 1.87 minutes in the BSS group and 7.69 ± 1.32 minutes in the Healon group. Surgery time was significantly shorter in the BSS group (*p* = 0.004).

## 4. Discussion

Cataract surgery requires a high level of skill and care in order to avoid any endothelial damage, which could lead to severe impairment of vision postoperatively. Bullous keratopathy after traumatic cataract surgery has been a major indication for keratoplasty in the past [28,29]. The use of viscoelastics during phacoemulsification has increased the safety profile of the procedure, reducing the endothelium-related complications [30]. Within this context, the aim of our study was to investigate whether the use or not of viscoelastics may affect the clinical outcomes after phacoemulsification performed by a highly experienced cataract surgeon.

Additionally, the production of ultrasound energy is associated with heat generation that can result in thermal damage of corneal endothelium. Friction was identified as the main source of heat generation during the surgery. If we want to gain more complete information about the amount of the generated heat, we need to know the local distribution of velocity. The motion of a Newtonian fluid is described by the non-linear Navier Stokes equations and the continuity equation. Those equations were numerically solved with some simplifications, such as: self heating rate of the probe is negligible, a thin layer of incompressible fluid flow exists around the phacoemulsification probe, and frictional heat generation takes place in the entire anterior chamber. These calculations yielded the velocity and the temperature distributions. In these calculations it was assumed that during surgery, the phacoemulsification probe was surrounded by AH or BSS or Healon (Figure 5, Figure 6, Figure 7, Figure 8, Figure 9, Figure 10 and Figure 11). The results of the simulations showed that heating was minimal when Healon was used as irrigation fluid for all chosen heating rates ($\dot{Q}$ = 0.66 W; 1.2 W; 2.4 W; 3 W). Additionally, there was no significant rise in temperature inside the eye in the first ten seconds of the modeled surgery. The maximum increase in corneal surface temperature of 4 °C was obtained for $\dot{Q}$ = 3 W. For lower input power settings, no significant temperature rise on the corneal surface was observed in the simulation. On the other hand, when the anterior chamber was completely filled with fluid such as AH or BSS, the temperature rise was around 6 °C for 5 s at the center of the corneal endothelium, for minimal values of heating power. If the anterior chamber is filled with Healon, the temperature rises no more than 0.5 °C (Figure 7 and Figure 11). Particularly, when the probe is in contact with Healon, which has a viscosity five times higher than that of BSS, or water, this fluid suppresses any heat generation in the simulated domain. At 30 seconds, the temperature on the corneal endothelium was elevated at maximum up to 54 °C or 40 °C in AH or BSS, respectively, for maximum heating rate of $\dot{Q}$ = 3 W. Based on the modeling of the heating rate, it can be concluded that with higher input power there is a potential risk of damaging the corneal endothelium.

The simulated velocity fields of AH and BSS are very similar during the surgery process. The streamline is symmetric and diverges uniformly from the center of pupil to the surrounding area. From the velocity distributions (Figure 5 and Figure 8), we can obtain the velocity values within 0.1–1.6 mm/s. A potentially worrying problem is whether the redistribution of the velocity profile affects the increase in intraocular pressure (IOP). The corneal endothelium is the thinnest layer of the cornea, and is a relatively “leaky” layer due to its high porosity. The relevant role of fluid dynamics quantities as well as porosity and permeability of the trabecular meshwork and flow resistance of AH on endothelial cell damage, and consequently on IOP will be the object of interest in our further investigations.

Mean ECD was comparable in the BSS group and the Healon group at first postoperative day, 1 week and 4 weeks after surgery. Mean endothelial cell loss was 4.5% (106.2 ± 226 cells/mm^2^) in the BSS group and 5.3% (122.2 ± 207 cells/mm^2^) in the Healon group, which is in compliance with other studies reporting endothelial cell loss rate varying between 3.8 and 11.8% [14, 15, 16, 17].

Mean ECD is not the only factor influencing corneal endothelial function. The morphometric characteristics of the endothelial cells (endothelial cell size and cell shape) appear to represent a sensitive indicator of the corneal endothelial function [8]. Four weeks after cataract surgery, the number of hexagonal cells decreased by 3.5% in the BSS group and by 3.2% in the Healon group, but the difference in the decrease of hexagonal cells (pleomorphism) between the two groups was not significant (*p* = 0.05). Coefficient of variation of cell size (polymegathism) was approximately 1.8% in the BSS group and 2% in the Healon group (*p* = 0.8). These results are comparable with those reported by Koch et al., who documented a decrease of hexagonal cells of 2.6% and a coefficient of variation of 2.7% after phacoemulsification with the use of viscoelastics [31].

To our knowledge, our study is the first to compare BSS and Healon in terms of their effect on corneal endothelium during phacoemulsification. 

In our study, we did not observe significant difference in ECD and endothelial morphometric characteristics between the BSS and Healon group postoperatively. To achieve these results, it was important to have a deep enough anterior chamber and to perform a careful implantation of a posterior chamber IOL through 2.35 mm aperture without touching the corneal endothelium. It was found that there was no difference in endothelial cell count loss between the Healon and BSS groups with no statistical difference in EPT. However, the surgery time was significantly shorter in the BSS group. This fact, as well as the fact that the cataract surgery was performed by an experienced surgeon, may indicate that the protective effect of viscoelastic may play a lesser role. The postoperative IOP peaks after using viscoelastics is explained by a viscosity-dependent increasing trabecular outflow resistance due to obstruction of the aqueous drainage system [32,33,34]. Most often, the IOP peaks occur within the first 8 h postoperatively, and despite viscoelastics elimination from the anterior chamber, they cannot always be prevented [35]. Therefore, an intraoperative application of Carbachol could be an option to reduce the IOP in the first postoperative hours. The transitory IOP peak over 20 mmHg, which occurred in nine cases of the BSS group 6 h after the cataract surgery, normalized at the first postoperative day. The reason for this IOP peak could have been a small quantity of methyl cellulose as a lubricating agent in the injector. In the Healon group only five cases had an IOP peak over 20 mmHg 6 h after the cataract surgery, which normalized at the first postoperative day. Meticulous removal of Healon even behind the IOL and application of Carbachol at the end of surgery, were crucial for achieving these results. It was interesting to note that there were no significant differences in IOP between the groups. It can be concluded that a complete washout of the Healon has a positive effect on IOP. BSCVA pre- and postoperatively was comparable in both groups. Apart from the transient postoperative IOP peaks, no other complication occurred in both groups.

These results indicate that phacoemulsification with BSS and continuous anterior chamber irrigation instead of viscoelastics, could provide comparable results in terms of clinical outcome and safety, with the viscoelastics-assisted cataract surgery, if performed by a highly experienced cataract surgeon.

## 5. Conclusions

Numerical simulation and modeling of the heating rates with a finite element software package, including both fluid and heat flows, can provide valuable information on thermal exposure during the surgery. Phacoemulsification with the use of BSS instead of Healon could provide excellent postoperative clinical outcomes regarding the best spectacle-corrected visual acuity, the intraocular pressure and the endothelial cell density, if performed by a highly experienced cataract surgeon.

## Figures and Tables

**Table 1:** Preoperative endothelial cell area pattern and shape.

	AVG±SD	MAX	MIN	CV	6A
BSS-group	457.7 ± 252 µm^2^	942.6 µm^2^	170.1 µm^2^	34.7 ± 5.9	60.4%
Healon-group	452.3 ± 126 µm^2^	933.9 µm^2^	158.2 µm^2^	34.9 ± 5.1	58.8%

**Table 2:** The first postoperative day endothelial cell area pattern and shape.

	AVG ± SD	MAX	MIN	CV	6A
BSS-group	481.8 ± 211 µm^2^	1014.0 µm^2^	170.0 µm^2^	36.7 ± 6.0	57.9%
Healon-group	467.0 ± 116 µm^2^	962.3 µm^2^	150.0 µm^2^	35.5 ± 5.2	57.1%

## Data Availability

The data presented in this study are available on request from the authors, in particular the datasets are archived in the clinics treated. The data are not publicly available as they contain information that could compromise the privacy of the participants.

## Abbreviation

AC—anterior chamber	EPT—effective phaco time
AVE—average cell area	IOL—intraocular lens
AVG—average cell size	IOP—intraocular pressure
BCVA—best corrected visual acuity	LOCS—lens opacities classification system
BSCVA—best sphere corrected visual acuity	MAX—cell maximum area
BSS—balanced salt solution	MIN—cell minimum area
CD—cell density	SC—Schlemm’s canal
CV—coefficient of variation	SD—standard deviation
ECD—endothelial cell density	TM—Trabecular meshwork
ECL—endothelial cell loss	VA—visual acuity
ECM—endothelial cell morphology	6A—percent of hexagonal cell
ECS—endothelial cell size	
